# Nutrient patterns and the skeletal muscle mass index among Polish women: a cross-sectional study

**DOI:** 10.1038/s41598-019-55367-5

**Published:** 2019-12-12

**Authors:** Anna Danielewicz, Jakub Morze, Małgorzata Obara-Gołębiowska, Mariusz Przybyłowicz, Katarzyna E. Przybyłowicz

**Affiliations:** 1 Department of Human Nutrition, University of Warmia and Mazury in Olsztyn, Poland; ul. Słoneczna 45F, 10-718 Olsztyn, Poland; 2 Department of Psychology of Development and Education, University of Warmia and Mazury in Olsztyn, Poland; ul. Prawocheńskiego 13, 10-447 Olsztyn, Poland; 3 Department of Gynecology and Obstetrics, Provincial Specialist Hospital in Olsztyn, ul. Żołnierska 18, 10-561 Olsztyn, Poland

**Keywords:** Lifestyle modification, Nutrition, Epidemiology

## Abstract

Ageing involves significant changes in skeletal muscle mass and its functioning. This study aimed to identify the major nutrient patterns (NPs) present in a sample of adult Polish women and evaluate their associations with the skeletal muscle mass index (SMI). A cross-sectional study initially recruited 527 women, and a final analysis was carried out on 275 women aged 32–60 years. Nutrient intake was assessed using fourteen repetitions of 24-hour dietary recall. NPs were derived using principal component analysis. Associations between adherence to NPs and the SMI were evaluated using linear regression models. Three NPs were identified: ‘Animal Protein-Vitamins’, ‘Fibre-Plant Protein-Minerals’ and ‘Fats’. In the adjusted model, the upper tertile compared to the bottom tertile of the ‘Animal Protein-Vitamins’ NP was related to a higher SMI (β = 0.123 95% CI: 0.019; 0.227; *P* for 1-SD increase of NP score = 0.009). No associations between the SMI and the ‘Fibre-Plant Protein-Minerals’ and ‘Fats’ NPs were observed. Our results indicate that high adherence to animal product-rich patterns might be related to higher muscle mass in adult women. Research on the influence of dietary and nutrient patterns on the quality of muscle tissue may contribute to the setting of guidelines for nutritional protection of skeletal muscle with ageing and, consequently, dietary recommendations that would improve the quality of women’s lives at the later stage of life.

## Introduction

Ageing involves significant changes in body composition, especially in skeletal muscle mass, which is a major contributor to frailty in the elderly^1^. The aetiology of decreased muscle mass has a multi-factorial basis that may include genetic heritability, atherosclerosis, insulin resistance, changes in endocrine function, decreases in protein synthesis, inflammation and nutritional deficiencies^2^.

Currently, a decrease in muscle mass, separately or in the context of sarcopenia risk, is usually assessed in people over 65 years of age. However, many experts claim that this problem should be considered in younger age groups^3^, especially women. Hormonal changes occurring during the menopausal transition result in a greater muscle mass decline in women than men. The advanced decrease in the concentrations of sex steroids after the menopausal transition may be an important factor in explaining the higher prevalence of low muscle mass among women aged between 60 and 70^4^. Loss of muscle mass and strength among postmenopausal women is often linked to a higher risk of non-communicable diseases, as well as osteoporosis, bone fractures and the progression of functional disability, which may contribute to frailty and decreased quality of life in the elderly^5^.

The menopause transition is also associated with lifestyle changes^6^. Common nutrition mistakes, poor compliance to dietary recommendations, along with low physical activity and a sedentary lifestyle^7^ may contribute to the increased decline in muscle mass and affect the health burden in further life^6,8^. A reduced intake of nutrients (especially protein, calcium and vitamin D)^9–12^ and lowered capacity for dietary anabolic response, most likely stimulated by hormonal and immunological changes^13,14^, are potential links between nutrition and loss of muscle mass. Most observational and interventional nutrition studies focused on the association between single nutrients (e.g., protein, polyunsaturated fatty acids, vitamins, minerals, and antioxidants) and skeletal muscle quality^9–11,15^. However, those studies provided ambiguous results, probably due to neglecting the synergistic effect of various components of a diet^16^. Therefore, an analysis of dietary patterns is potentially of great importance for the optimisation of proper skeletal muscle mass. Dietary patterns are useful tools for the description and interpretation of dietary habits in nutritional epidemiology^17,18^. Although in previous studies, the hypothesis-driven and data-driven dietary patterns were based on the frequency or amount of food groups consumed^19^, nutrient-based patterns were not previously investigated in the context of muscle mass decrease. Considering synergistic nutrient intake by exploring nutrient patterns may help to identify such clusters.

It is important to understand these associations since the diet has a significant effect on health, and strong scientific evidence can provide the basis for creating intervention programmes in the future. Therefore, the aim of the present study was to identify the major nutrient patterns (NPs) present in a sample of adult Polish women and evaluate their associations with the skeletal muscle mass index (SMI).

## Results

A comparison of the basic characteristics of the included and excluded subjects is presented in Supplementary Table S1. Excluded women were younger (*P* = 0.043). Their daily energy intake was lower (*P* = 0.002) and dietary protein content was higher (*P* = 0.039) than those of the included participants. The final study sample included 275 women with a mean age of 47.1 years. The baseline characteristics of the participants among tertiles of SMI are presented in Table 1. Women in the bottom tertile of SMI were more likely to be taller, have lower body weight, BMI, WC, WtHR, MUAC, ASM, and amount and content of FM, and have higher economic status. There were no differences between the menopausal status, place of residence and educational level among tertiles of SMI.

**Table 1:** Anthropometric parameters and demographic characteristics by bottom and upper tertiles of skeletal muscle mass index.

Characteristics	Total		Skeletal Muscle Mass Index						*P*
			Bottom tertile		Middle tertile		Upper tertile		
*n*	275		90		93		92		
Age, years^a^	47.1	6.0	46.4	5.3	47.2	6.2	48.0	6.3	0.473
Weight, kg	65.2	59.0; 74.0	58.0	54.0; 60.0	65.0	62.0; 68.7	79.7	71.0; 93.4	<0.001
Height, m^a^	160.0	5.3	161.7^†^	5.7	160.2	4.7	160.0^†^	5.2	0.038
BMI, kg/m^2^	25.6	22.8; 28.8	22.0	20.7; 23.0	25.6	24.6; 26.4	31.3	28.7; 35.1	<0.001
WC, cm	81.0	73.0; 90.0	71.0	68.0; 75.0	81.0	74.0; 85.0	95.3	87.0; 102.0	<0.001
WtHR	0.5	0.5; 0.6	0.4	0.4; 0.5	0.5	0.5; 0.5	0.6	0.6; 0.6	<0.001
MUAC, cm	20.9	18.2; 23.0	19.0^†^	17.0; 21.3	21.0	19.9; 22.8	22.5^†^	19.6; 26.0	<0.001
FM, kg	21.4	17.8; 26.2	16.4	14.7; 19.4	21.1	18.5; 22.9	29.2	24.6; 34.8	<0.001
%FM, %	32.7	29.1; 36.3	28.9	26.7; 33.2	31.5	29.3; 34.3	36.5	33.3; 38.8	<0.001
ASM, kg	20.8	19.1; 22.5	18.8	17.8; 19.7	20.8	19.5; 21.3	24.0	21.9; 27.3	<0.001
Menopause									
yes	55	20.0	13	13.8	19	20.4	23	25.0	0.156
no	220	80.0	81	86.2	74	79.6	69	75.0	
Economic status									
low	7	2.6	1	1.1	3	3.3	3	3.3	0.003
medium	129	46.9	30	33.3	56	60.2	43	46.7	
high	139	50.5	59	65.6	34	36.7	46	50.0	
Place of residence (thousand citizens)									
village	84	30.5	29	32.2	24	25.8	31	33.7	0.068
<50	57	20.7	13	14.4	30	32.3	14	15.2	
50–100	29	10.5	11	12.2	9	9.7	9	9.8	
>100	105	38.2	37	41.1	30	32.3	38	41.3	
Education level									
primary/vocational	59	21.5	13	14.4	23	24.7	23	25.0	0.002
high/technical	111	40.4	30	33.3	34	36.6	47	51.1	
higher education	105	38.2	47	52.2	36	38.7	22	23.9	

Note: Values are presented as presented median and interquartile (IQR) or number (%) unless other indicated ^a^Values presented as mean and standard deviation (SD). BMI, body mass index; WC, waist circumference. WtHR, waist-to-height ratio; MUAC, mid-upper-arm muscle circumference; FM, total body fat mass; %FM, percentage of total body fat mass; ASM, Appendicular Skeletal Muscle Mass. *P* was obtained using Kruskal-Wallis with Dunn’s post-hoc test or ANOVA with Tukey’s post-hoc test for continuous variables and chi-square or Fisher’s exact test for categorical variables. ^†^Differences between bottom and upper tertile mean.

Women did not differ in daily energy intake between tertiles of SMI (Table 2). Protein intake (% energy) was significantly higher in the upper tertile than that in the middle tertile. However, there were no differences in carbohydrate and fat intake (% energy). Energy-adjusted total and animal protein intakes, as well as the animal-to-plant protein ratio, were higher in the upper tertile compared to those in the bottom or middle tertiles. Energy-adjusted phosphorus and vitamin B2 intakes were significantly higher in the upper tertile when compared to intakes in the middle tertile but not when compared to the bottom. Intakes of other macro- and micronutrients revealed no differences. However, taking into account the requirements of nutrient intake, women in the upper tertile of SMI compared to the bottom tertile were more likely to have an inadequate intake of protein (32.6% vs. 12.2%) and were less likely to have an inadequate intake of calcium (55.4% vs. 76.7%), potassium (94.6% vs. 98.9%), vitamin A (12.0% vs. 30.0%), niacin (10.9% vs. 27.8%) and vitamin B6 (8.7% vs. 23.3%) (Supplementary Table S2).

**Table 2:** Daily energy and nutrients intake by bottom and upper tertiles of skeletal muscle mass index.

Nutrients	Total		Skeletal Muscle Mass Index						*P*
			Bottom tertile		Middle tertile		Upper tertile		
	Me	IQR	Me	IQR	Me	IQR	Me	IQR	
*n*	275		90				92		
Energy, kcal	1651.2	1366.9; 1953.2	1672.1	1285.4; 1920.6	1599.5	1351.0; 1916.8	1663.1	1407.0; 1974.9	0.211
Protein, % E	15.4	14.3; 17.0	15.4	14.2; 16.7	15.0^‡^	14.0; 16.3	15.9^‡^	14.7; 17.9	0.021
Fat, % E	37.8	4.5	37.7	4.0	38.0	37.1; 38.9	38.0	5.0	0.870
Carbohydrates, % E	46.4	5.0	46.8	5.0	46.6	45.5; 47.7	46.0	5.0	0.621
Macronutrients*									
Total protein, g	73.7	68.5; 81.7	73.3	68.5; 79.9	72.5^‡^	67.5; 78.2	76.5^‡^	70.7; 85.6	0.019
Animal protein, g	48.7	43.5; 55.9	47.7^†^	43.2; 55.0	47.5^‡^	42.4; 53.2	51.5^†‡^	45.9; 60.2	0.005
Plant protein, g	24.8	22.9; 27.0	25.6	23.4; 27.8	24.6	22.5; 26.9	24.7	22.7; 26.5	0.215
Animal-Plant Protein Ratio	2.0	1.7; 2.3	1.9^†^	1.6; 2.2	2.0	1.7; 2.3	2.1^†^	1.8; 2.5	0.015
Total Fat, g	80.7	9.7	80.5	9.0	81.2	79.2; 83.3	81.0	10.0	0.826
Cholesterol, mg	397.5	321.4; 472.6	393.9	314.2; 488.0	382.2	326.2; 442.6	408.3	324.5; 493.5	0.515
Carbohydrates, g	242.6	24.3	244.2	24.0	243.2	238.1; 248.4	240.0	24.0	0.712
Fiber, g	19.2	17.2; 21.8	19.2	17.4; 21.8	19.1	17.4; 21.2	19.3	16.7; 22.3	0.921
Micronutrients*									
Calcium, mg	630.0	533.5; 732.1	633.9	562.3; 740.2	593.2	520.9; 702.6	648.3	550.4; 755.0	0.083
Phosphorus, mg	1174.6	1090.6; 1288.7	1192.4	1111.5; 1294.4	1149.8^‡^	1042.1; 1268.2	1199.7^‡^	1110.9; 1331.0	0.026
Magnesium, mg	272.9	247.5; 304.5	275.1	247.5; 305.2	272.9	248.8; 289.4	266.3	247.0; 314.7	0.609
Iron, mg	12.1	11.0; 13.2	12.1	10.6; 13.7	11.9	11.1; 12.7	12.3	11.2; 13.2	0.585
Zinc, mg	10.0	9.2; 10.9	10.1	9.5; 10.8	9.8	9.2; 10.7	10.3	9.3; 11.3	0.132
Copper, mg	1.2	1.1; 1.3	1.2	1.1; 1.3	1.2	1.0; 1.3	1.2	1.0; 1.4	0.782
Potassium, mg	3152.9	2844.1; 3544.6	3099.7	2763.2; 3519.3	3180.0	2844.1; 3468.3	3161.7	2869.6; 3631.3	0.739
Vitamin A, µg	804.7	661.9; 1079.4	781.1	636.9; 1116.8	772.2	653.8; 985.7	853.3	720.9; 1082.2	0.132
Vitamin E, mg	10.5	8.5; 12.4	10.6	8.2; 12.8	10.5	8.3; 12.5	10.4	8.7; 11.9	0.752
Vitamin B1, mg	1.1	1.0; 1.3	1.2	1.0; 1.3	1.1	1.0; 1.3	1.1	1.0; 1.2	0.436
Vitamin B2, mg	1.5	1.3; 1.7	1.5	1.3; 1.6	1.4^‡^	1.3; 1.6	1.5^‡^	1.4; 1.7	0.021
Niacin, mg	17.3	14.9; 19.4	17.3	14.4; 19.1	17.2	15.2; 19.4	17.5	14.9; 20.0	0.332
Vitamin B6, mg	1.8	1.6; 2.0	1.8	1.6; 2.0	1.8	1.6; 2.0	1.8	1.6; 2.2	0.487
Vitamin C, mg	45.4	34.6; 57.2	45.5	34.1; 56.6	44.7	37.6; 59.4	46.5	33.4; 54.9	0.813

Note: Values are presented as presented median (Me) and interquartile range (IQR) or number (%), unless other indicated. E, Energy. *P* was obtained using Kruskal-Wallis with Dunn’s post-hoc test or ANOVA with Tukey’s post-hoc test. ^a^Values presented as mean and standard deviation (SD). *Daily dietary nutrient intake adjusted for energy (2000 kcal). ^†^Differences between bottom and upper tertile, ^‡^Differences between middle and upper tertile.

Three NPs were derived and explained 59.8% of the total variance. The first, identified as ‘Animal Protein-Vitamins’, explained 35.1% of the variance and was characterized by high intakes of vitamin B_2_ (0.88), animal protein (0.74), phosphorus (0.70), zinc (0.69), iron (0.66), niacin (0.65), vitamin B_6_ (0.58), vitamin A (0.57) and potassium (0.53). The second, identified as ‘Fibre-Plant Protein-Minerals’, explained 15.4% of the variance and was characterized by high intakes of fibre (0.83), magnesium (0.76), cooper (0.74), plant protein (0.69), potassium (0.63) and vitamin E (0.52), as well as low intake of cholesterol (−0.58). The third, identified as ‘Fats’, explained 9.3% of the variance and was characterized by high intakes of total fat (0.89) and vitamin E (0.55), as well as low intake of carbohydrates (−0.91). The factor loadings for the identified NPs are presented in Table 3.

**Table 3:** Factor loadings of identified nutrient patterns.

Variables	Nutrient Patterns		
	Animal Protein - Vitamins	Fiber-Plant Protein-Minerals	Fats
Vitamin B_2_	**0.88**	−0.03	−0.09
Animal Protein	**0.74**	−0.14	0.30
Phosphorus	**0.70**	0.37	0.18
Zinc	**0.69**	0.32	0.25
Iron	**0.66**	0.30	−0.02
Niacin	**0.65**	0.25	0.30
Vitamin B_6_	**0.58**	0.48	0.13
Vitamin A	**0.57**	−0.07	−0.11
Potassium	**0.53**	**0.63**	0.03
Fiber	0.21	**0.83**	−0.20
Magnesium	0.43	**0.76**	0.13
Cooper	0.42	**0.74**	0.07
Plant Protein	−0.02	**0.69**	−0.14
Cholesterol	0.28	**−0.58**	0.12
Vitamin E	−0.10	**0.52**	**0.55**
Carbohydrate	−0.27	0.21	**−0.91**
Total Fat	−0.06	−0.27	**0.89**
Calcium	0.42	0.16	−0.07
Vitamin B_1_	0.36	0.45	0.33
Vitamin C	0.18	0.40	−0.09
Variance explained (%)	35.1	15.4	9.3

Note: Bold are factor loadings of ≥|0.5| included in identified factors. Total explained variance was 59.8%.

Associations between tertiles of NPs and the SMI are shown in Table 4. The upper tertile of the ‘Animal Protein-Vitamins’ pattern was related to a higher SMI in Model 1 (β = 0.158 95% CI: 0.022; 0.293). This effect remained significant when controlling for age, energy intake and %FM in Model 2 (β = 0.129 95% CI: 0.024; 0.234) and when further controlling for economic status and place of residence in Model 3 (β = 0.123 95% CI: 0.019; 0.227). Moreover, an increase of 1-SD in the ‘Animal Protein-Vitamins’ NP score was linearly associated with the SMI in the crude (*P* = 0.016) and adjusted models (*P* = 0.005 and *P* = 0.009, respectively). No associations with SMI were observed for the ‘Fibre-Plant Protein-Minerals’ and ‘Fats’ NPs. Post hoc power analysis indicated that the power to detect associations between adherence to NP score and the SMI was greater than 99% for all adjusted regression models.

**Table 4:** Associations between adherence to nutrient patterns and skeletal muscle mass index.

Nutrient Patterns	Tertiles of Nutrient Patterns						1-SD increase of NP score	
	Bottom		Middle		Upper			
	β (95% CI)	*P*	β (95% CI)	*P*	β (95% CI)	*P*	β (95% CI)	*P*
**Animal Protein – Vitamins**								
*n* (NP score)	92 (−2.46 to −0.46)		92 (−0.45 to 0.26)		91 (0.27 to 4.94)			
Model 1^a^	Ref.		0.027 (−0.109; 0.162)	0.705	0.158 (0.022; 0.293)	0.023	0.144 (0.026; 0.262)	0.016
Model 2^b^	Ref.		0.059 (−0.044; 0.162)	0.257	0.129 (0.024; 0.234)	0.016	0.132 (0.039; 0.225)	0.005
Model 3^c^	Ref.		0.051 (−0.050; 0.152)	0.302	0.123 (0.019; 0.227)	0.020	0.122 (0.030; 0.215)	0.009
**Fiber-Plant Protein-Minerals**								
*n* (NP score)	92 (−2.20 to −0.45)		92 (−0.43 to 0.34)		91 (0.34 to 3.87)			
Model 1^a^	Ref.		−0.060 (−0.197; 0.077)	0.387	−0.057 (−0.194; 0.080)	0.410	−0.062 (−0.007; 0.244)	0.298
Model 2^b^	Ref.		−0.054 (−0.158; 0.049)	0.304	−0.039 (−0.146; 0.067)	0.463	−0.044 (−0.137; 0.050)	0.359
Model 3^c^	Ref.		−0.044 (−0.148; 0.060)	0.410	−0.070 (−0.177; 0.037)	0.195	−0.065 (−0.159; 0.028)	0.170
**Fats**								
*n* (NP score)	91 (−2.44 to −0.48)		93 (−0.47 to 0.35)		91 (0.37 to 3.25)			
Model 1^a^	Ref.		0.127 (−0.009; 0.264)	0.067	0.006 (−0.130; 0.143)	0.929	0.058 (−0.060; 0.177)	0.337
Model 2^b^	Ref.		0.068 (−0.038; 0.173)	0.207	−0.005 (−0.110; 0.100)	0.924	0.042 (−0.049; 0.133)	0.362
Model 3^c^	Ref.		0.070 (−0.034; 0.174)	0.188	−0.001 (−0.104; 0.102)	0.988	0.051 (−0.038; 0.141)	0.261

Note: Results from linear regression analyses are presented as β coefficient and 95% confidence interval (95% CI). NP, Nutrient pattern; SD, standard deviation. ^a^Model 1 was crude; ^b^Model 2 was adjusted for age (years), energy intake (kcal/d) and total body fat mass (%); ^c^Model 3 was adjusted for age (years), energy intake (kcal/d) and total body fat mass (%), economic status (low/medium/high) and place of residence (village, a city with a population less than 50 thousand citizens, a city with a population of 50–100 thousand citizens or city with a population of more than 100 thousand citizens).

## Discussion

Although there are a number of studies exploring the relationships between diet and the SMI, the present study is unique in that it evaluated the impact of data-driven nutrient patterns on skeletal muscle mass in a group of adult Polish women. The main finding showed that high adherence to the ‘Animal Protein-Vitamins’ NP was associated with a higher SMI.

A comparison of our results with previous findings is challenging because of the differences in the studied population, age groups, dietary habits and method of muscle mass assessment. Moreover, previous studies focused predominantly on evaluating food-based dietary patterns or single nutrient associations^10,11,15,20,21^. While nutrient-based patterns is a novel approach in the context of the SMI, our findings need to be interpreted in terms of NP components. Nutrients characterized by derived patterns might represent the diversity of the diet in the studied sample. The components included in the ‘Animal Protein-Vitamins’ NP may suggest high consumptions of meat, offal, fish, dairy products, potatoes and yellow-orange vegetables. High adherence to the ‘Fibre-Plant Protein-Minerals’ NP may be associated with high consumption of vegetables, fruits, grains, and legumes and low consumption of animal products. The ‘Fats’ NP may be characterised by consumption of oil, olives, margarine and butter.

The results of Chan *et al*.’s^22^ study showed no association between the ‘Vegetable-Fruits’, ‘Snacks-Drinks-Milk Products’ and ‘Meat-Fish’ dietary patterns and the risk of sarcopenia among community-dwelling older women. Additionally, no relationship with the incidence of sarcopenia after the 4-year follow-up was detected. One of the dietary patterns widely referenced in the context of muscle mass and sarcopenia is a Mediterranean diet. Two cross-sectional studies reported that this diet may reduce the risk of sarcopenia by up to 60%^20,21^. The high adherence to the Mediterranean diet was correlated with higher muscle mass indexes^21,23,24^ and also in women under 62 years^23,24^. However, not all studies confirm this association^20,21^. This result may be explained by the fact that the Mediterranean diet is rich in antioxidants, which can reduce the negative effects of oxidative stress, perceived as one of the main mechanisms in the pathogenesis of muscle mass loss due to age^15,25^. An increase in reactive oxygen species and blunted antioxidant defences leads to the mutation and dysfunction of mitochondrial DNA. Overexpression of antioxidants may protect against oxidative damage to mitochondrial respiration and ATP production in skeletal muscle^5^. Additionally, products with an alkalizing effect (e.g., as found in the Mediterranean diet) may have a protective effect on the fat-free body mass by reducing the acid-forming effect of a high protein supply, which is necessary for muscle regeneration^26,27^. Some of the previously mentioned studies, parallel to the Mediterranean diet, evaluated the Western or Mixed dietary pattern and showed no association with the risk of sarcopenia^20,21^, and surprisingly, stronger adherence to both dietary patterns was associated with a higher muscle mass^20^. A high-fat diet could indicate an increase in the risk of low muscle weight by triggering an inflammatory condition of the body and promoting atherosclerosis^28^.

In our study, the median protein intake was higher in the upper tertile of SMI in comparison with that in women in the bottom tertile. Moreover, women in the upper tertile consumed more animal protein than other groups, whereas no difference was observed for plant protein intake. An important insight from our study is that the ratio of animal-to-plant protein intake was higher among women with a higher SMI. These results may suggest that the source of dietary protein is no less important than the amount of consumption. In the Polish population, a major source of protein is animal products. These include meat, fish and dairy, which are a great source of leucine. Leucine is a crucial amino acid for muscle protein synthesis, regulation of the mTOR pathway and protection from protein degradation^5^. However, in a study of the Taiwan population, the intake of plant protein was positively correlated with muscle mass. This effect might be explained by the high intake of legumes, including soy and cowpeas, which provide a sufficient supply of leucine^10^. These findings suggest that, apart from the amount or source of protein, the amino acid matrix may also be crucial in protecting against age-related muscle loss. Genaro *et al*.^29^ found no differences between muscle mass and consumption of total, animal, plant or energy-adjusted protein intake. However, when considering protein intake as the amount per kg of body weight, a significantly higher muscle mass was recorded in women consuming >1.2 g/kg/d in comparison to that in women consuming <0.8 g/kg/d^29^. Moreover, Scott *et al*.^14^ demonstrated that the intake of protein and its supply after 2.6 years in comparison to the initial state was positively related to the ALM value. Additionally, as shown by Mamerow *et al*.^30^, the distribution of protein supply across three meals during a day resulted in a 25% growth in muscle protein synthesis in comparison to an unequal supply throughout the day. Nevertheless, our study did not involve an analysis of the meal’s consumption patterns.

In our results, minerals and vitamins were identified in all three NPs. Previous studies reported a positive correlation between ALM and fibre, niacin, potassium, zinc^14^, magnesium^14,31^, calcium^11,14^ and phosphorus^14,32^, as well as a negative correlation with vitamin A^14^. Although Chaput *et al*.^33^ observed no difference in the vitamins A, C and E intake between sarcopenic and nonsarcopenic subjects, they found a significantly higher percentage of inadequate intake of those vitamins in a group with sarcopenia. In the presence of multiple differences in nutrient intake, NPs might be a more suitable method to capture their overall effect on muscle mass.

On the basis of the research conducted, it is suggested that the intake of a wide range of food products, ensuring an appropriate supply of necessary nutrients related to the maintenance of proper muscle mass, is important in the prevention and treatment of muscle mass loss^14^. Hormonal disorders that occur with age might be considered a breakthrough moment in muscle mass loss^26^ and must be considered as a point for the implementation of preventive strategies, which can benefit in lowering the risk of sarcopenia among the elderly. A multi-dimensional approach to life quality, including diet, physical activity, stress level and environmental factors, may provide the basis for formulating recommendations for the delay in the occurrence and slowing down the progression of sarcopenia at various life stages.

There are several limitations pertaining to this study. First, the use of the Lee equation^34^ to estimate muscle mass may overestimate or underestimate its real value. However, this method was validated on a large and diversified population with regard to the gold standard methods, i.e., magnetic resonance imaging and dual-energy X-ray absorptiometry, where good concordance rates were obtained^35^. Second, most of the studies, with regard to the analysed research problem, chose elderly women aged 65 and older. Consequently, the obtained results must be carefully compared to the outcome of other studies due to the age of women included in our study. However, it could also be a strength of the study because this age group of women is particularly exposed to muscle mass loss as a result of changes in endocrine function during menopause. The third limitation of the study, which may reduce the area of its inference, is the lack of muscle strength and physical performance assessment. Therefore, a separate assessment of the muscle mass allows only a partial interpretation of results in the context of overall sarcopenia risk. Another limitation of our study is the method applied for gathering nutritional data. The 24-hour dietary recall method depends on the respondent’s memory and the skills of the interviewer. A potential strength of this method in our study is the fourteen repetitions of the interview at irregular time intervals, which might eliminate variability related to memory and seasons of the year and, as result, improve the quality of dietary intake estimates^36,37^. A limitation that might affect the conclusions was the number of subjective choices made using PCA to derive NPs. These included (1) the use of energy-adjusted nutrient intakes, (2) selection of the number and factor rotation technique and (3) the decision on the factor loading cut-off point. Moreover, this method does not allow for the division of the examined sample into separated groups but only permits an evaluation of adherence to a specific pattern among the subjects^38^. Finally, the nature of a cross-sectional study does not allow us to determine a causal link between diet and muscle mass loss, but it may indicate crucial insights for designing future dietary interventions.

This study is the first in Central and Eastern Europe to investigate the association between nutrient patterns and low muscle mass in adult women. Stronger adherence to a NP correlated with animal protein and vitamins was associated with higher muscle mass, while no association was observed for a NP correlated with fibre, plant protein, minerals and fats. Further research is needed to fill the gaps in dietary recommendations for women to delay age-related skeletal muscle wasting.

## Methods

### Participants

**Figure 1:**
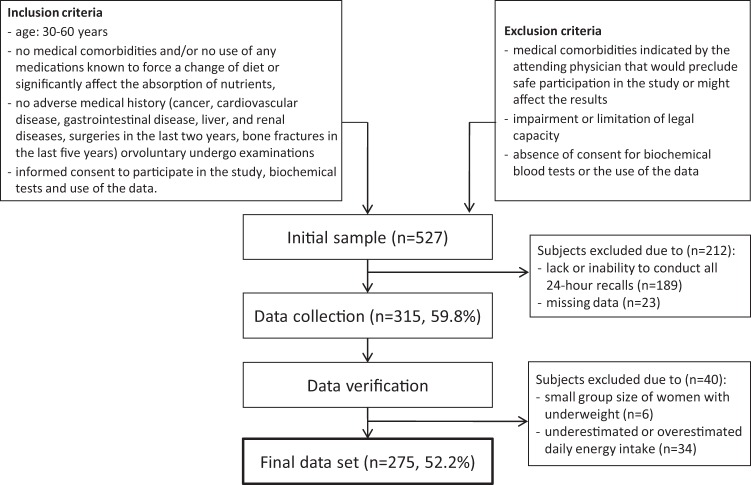
Flowchart of data collection.

A cross-sectional study with the snowball sampling method was carried out in 2009–2014 in the province of Warmia and Mazury in Poland. The respondents were white women (Caucasian ethnicity group) who volunteered to participate in the study on the basis of information obtained from general practitioners, women’s organisations and local government authorities. Women were eligible for study inclusion if they were aged 30–60 years, had no medical comorbidities and/or no use of any medications known to force a change in diet or to significantly affect the absorption of nutrients, and had no adverse medical history (cancer, cardiovascular disease, gastrointestinal disease, liver, and renal diseases, surgeries in the last two years, bone fractures in the last five years) or other medical comorbidities indicated by the attending physician that would preclude safe participation in the study or might affect the results. Other inclusion criteria included willingness to undergo examinations, expressing consent for biochemical tests and the use of the data, expressing informed consent to participate in the study, and a lack of impairment or limitation of legal capacity. Initially, 527 women were recruited, and 275 (52.2%) individuals were enrolled in the final sample. Women were excluded from the study due to the inability to perform all fourteen 24-hour dietary recalls (35.9%), missing data (4.4%), underestimated or overestimated daily energy intake (6.5%) and being underweight (1.1%) (Fig. 1).

The Bioethical Committee at the Warmia and Mazury Chamber of Physicians by Resolution No. 14/2003/II granted its approval to conduct the study. All experiments were performed in accordance with relevant guidelines and regulations, including the Declaration of Helsinki for Medical Research Involving Human Subjects, and all the participants provided written informed consent to undergo examinations.

### Dietary data collection

Dietary data were collected face-to-face by trained interviewers in the place of residence of participants, using the fourteen repetitions of the 24-hour dietary recall method^39^ at irregular intervals (within 1 month). To assess portion sizes, the ‘Album of Photographs of Food Products and Dishes’ was used^40^. The album contained photographs of 201 life-size food items commonly consumed in Poland, in three different portion sizes. Portion sizes were estimated by participants and then calculated into the edible portion. The average daily intake of energy and nutrients was calculated based on the Polish table of food composition using Dieta 4.0 software (The National Food and Nutrition Institute: Warsaw, Poland)^41^. Inadequate nutrient intakes were assessed using the cut-off point method^42^ in which an estimated average requirement (EAR) and adequate intake (AI) were established according to Polish dietary recommendations (Supplementary Table S3)^43^. Energy intake data were verified according to McCrory *et al*.’*s*^44^ method, which allows for the identification of underestimated or overestimated daily energy intake (<800 kcal and >4000 kcal). We used the density method to adjust the data obtained for energy per 2000 kcal to allow comparison of nutrient intake among the participants. Energy-adjusted data provided the basis for further analysis.

### Anthropometry and body compositions

Body weight was measured using an electronic scale to an accuracy of 0.1 kg, and the results were adjusted by 0.5 kg due to measurements taken while the subject was wearing light clothing. Body weight was measured at various times of the day, without the fasting state requirement. Body height was determined with an anthropometer, with an accuracy of 0.5 cm. Waist circumference was measured with flexible, non-elastic tape at the smallest abdominal girth between the lowest rib and the upper anterior iliac spine, with an accuracy of 0.1 cm. For weight, height and waist circumference, the mean of two measurements was taken. Skinfold thickness was measured using a calliper with an accuracy of 0.2 mm and measured at the triceps, biceps, subscapular and suprailiac sites. The mean of three measurements was taken^39^. Based on the obtained data, the following anthropometric parameters were calculated: body mass index (BMI), waist-to-height ratio (WtHR), mid-upper arm circumference (MUAC) and total fat mass (FM) and content (%FM). BMI was calculated as weight in kilograms (kg) divided by height in metres squared (m^2^) and evaluated pursuant to the World Health Organization (WHO) criteria^45^. The WtHR was calculated as the waist circumference in centimetres divided by height in centimetres. MUAC (the size of the muscle mass and an index of protein reserves) was calculated using the following equation: MUAC = arm circumference (cm) – π x triceps skinfold (cm)^46^. The body fat content was calculated using body density estimated from the skinfold thickness based on Durnin & Womersley’s algorithm^47^ and Siri’s equation^48^.

Muscle mass was estimated by the appendicular skeletal muscle mass (ASM) based on the Lee equation^31^: ASM = 0.244 × body weight (kg) + 7.8 × height (m) + 6.6 × sex − 0.098 × age (years) + race − 3.3, (for women R^2^ = 0.90, P < 0.05 and SEE = 2.8 kg)^49^. For sex, 0 denoted female and 1 denoted male. For ethnicity groups, 1.2 was used for Asian, 1.4 for African American, and 0 for white and Hispanic. ASM was adjusted by the height squared to obtain an index of relative skeletal muscle mass (SMI; kg/m2). Insertion of the square of height in the denominator provides minimizing of the correlation of the SMI with height across different study populations^50^.

The occurrence of menopause was determined on the basis of the menstrual cycle regularity, and postmenopause was defined as an absence of a period for the last twelve months or more. To describe socio-economic status, women were asked to identify their economic status (as low, medium or high), place of residence (as a either a village, a city with a population of less than 50 thousand citizens, a city with a population of 50–100 thousand citizens or a city with a population of more than 100 thousand citizens) and education level (as primary/vocational school, high/technical school or higher education).

### Statistical analysis

The baseline study characteristics and energy-adjusted nutrient intakes were summarized according to tertiles of SMI. The normality assumption was checked using the Shapiro-Wilk test. Continuous variables are presented as the mean and standard deviation (SD) if they followed a normal distribution or median and interquartile range (IQR) in cases of a skewed distribution. Differences between SMI tertiles were evaluated using the analysis of variance (ANOVA) with Tukey’s post hoc test or, when not normally distributed, with the Kruskal-Wallis test and Dunn’s post hoc test. Categorical variables are expressed using the number and percentage and compared between tertiles using the chi-square or Fisher’s exact test.

Principal component analysis (PCA) was implemented to derive NPs. The input data had a continuous form and included a correlation matrix of 20 energy-adjusted nutrients (animal protein, plant protein, total fat, cholesterol, carbohydrates, fibre, calcium, phosphorus, magnesium, iron, zinc, copper, potassium, and vitamins: A, E, B1, B2, niacin, B6 and C). Assumptions for the PCA were checked by calculating the Kaiser-Meyer Olkin (KMO) index and performing Bartlett’s test for sphericity. The KMO value was 0.924, and Barlett’s test was significant at *p* < 0.001. The number of factors was determined using the scree plot method and Kaiser’s criterion (Eigenvalue ≥1.00). The normalized varimax method was applied to provide orthogonal rotation and maximize the dispersion of loadings within factors. For each factor, nutrients with a loading coefficient of ≥|0.5| were identified as a gauge of the substantive importance of a given variable to a given factor. Then, NP scores representing individual participant adherence to the pattern were calculated by multiplying the factor loadings and nutrient intakes. NPs were labelled using nutrients that were positively loaded on the factor. NP scores were further divided into tertiles.

To determine associations between tertiles of NPs and the SMI, linear regression models were fitted. The bottom tertile of each pattern was used as a reference group. The SMI was transformed using the Box-Cox method for the analysis due to the skewness of the distribution. Three adjustment sets were considered: Model 1 – crude; Model 2 – adjusted for age, energy intake and %FM (all as continuous variables); and Model 3 – Model 2 plus economic status and place of residence (as categorical variables). Additionally, regression models (both crude and adjusted) with the per standard deviation increase in the NP score as an independent variable were fitted. For these models with adjustment for both covariate sets (Models 2 and 3), we calculated the post hoc statistical power of the observed effects. A *P* value < 0.05 was considered significant in all tests. The statistical analysis was conducted using STATISTICA software (version 13 PL; StatSoft Inc.; Krakow, Poland).

## Supplementary information

Supplementary material

## Data Availability

The datasets generated during and/or analysed during the current study are available from the corresponding author on reasonable request.
