# Supplementary material for: Nutrient patterns and the skeletal muscle mass index among Polish women: a cross-sectional study
Source: Sci Rep. 2019 Dec 12;9:18930. doi: 10.1038/s41598-019-55367-5 (PMC6908721; doi:10.1038/s41598-019-55367-5)
Supplement: Supplementary file 1 — Supplementary material [file 41598_2019_55367_MOESM1_ESM.docx]

Nutrient patterns and the skeletal muscle mass index among Polish women: a cross-sectional study

Anna Danielewicz^1,*^, Jakub Morze^1^, Małgorzata Obara-Gołębiowska^2^, Mariusz Przybyłowicz^3^, Katarzyna E. Przybyłowicz^1^

^1^ Department of Human Nutrition, University of Warmia and Mazury in Olsztyn, Poland; ul. Słoneczna 45F, 10-718 Olsztyn, Poland

^2^ Department of Psychology of Development and Education, University of Warmia and Mazury in Olsztyn, Poland; ul. Prawocheńskiego 13, 10-447 Olsztyn, Poland

^3^ Department of Gynecology and Obstetrics, Provincial Specialist Hospital in Olsztyn, ul. Żołnierska 18, 10-561 Olsztyn, Poland

***** anna.danielewicz@uwm.edu.pl

**SUPPLEMENTARY MATERIAL**

| **Characteristics** | **Participants** | | | | | ***P*** |
| --- | --- | --- | --- | --- | --- | --- |
|  | **Included** | |  | **Excluded** | |  |
| *n* | 275 | |  | 231 | |  |
| Age, years | 47 | (43; 52) |  | 46 | (42; 50) | **0.043** |
| Weight , kg | 65.2 | (59.0; 74.0) |  | 66.0 | (58.0; 74.0) | 0.100 |
| Height, m | 160.5 | (157.0; 164.0) |  | 161.0 | (157.5; 166.0) | 0.740 |
| BMI, kg/m^2^ | 25.6 | (22.8; 28.8) |  | 25.1 | (22.7; 28.6) | 0.259 |
| WC, cm | 81.0 | (73.0; 90.0) |  | 80.0 | (73.5; 89.0) | 0.623 |
| WtHR | 0.5 | (0.5; 0.6) |  | 0.5 | (0.5; 0.5) | 0.284 |
| MUAC, cm | 20.9 | (18.2; 23) |  | 20.6 | (18.8; 22.2) | 0.195 |
| FM, kg | 21.4 | (17.8; 26.2) |  | 21.2 | (17.1; 25.6) | 0.380 |
| %FM, % | 32.7 | (29.1; 36.3) |  | 32.4 | (28.8; 35.2) | 0.114 |
| ASM, kg | 20.8 | (19.1; 22.5) |  | 20.9 | (18.9; 23.1) | 0.818 |
| SMI, kg/m^2^ | 8.0 | (7.4; 8.7) |  | 8.0 | (7.3; 8.7) | 0.453 |
| *n* | 275 | |  | 64 | |  |
| Energy, kcal/day | 1688.5 | (1378.8; 2008.0) |  | 1355.1 | (855.7; 2051.3) | **0.002** |
| Protein, % energy | 15.4 | (14.3; 17.0) |  | 16.2 | (14.9; 18.2) | **0.039** |
| Fat , % energy | 37.7 | (35.1; 41.0) |  | 35.3 | (33.7; 39.8) | 0.053 |
| Carbohydrates, % energy | 46.6 | (43.1; 50) |  | 47.1 | (43; 50.6) | 0.516 |
| *n* | 275 | |  | 243 | |  |
| Menopause |  |  |  |  |  |  |
| yes | 55 | (20.0) |  | 33 | (13.6) | 0.052 |
| no | 220 | (80.0) |  | 210 | (86.4) |  |
| *n* | 275 | |  | 108 | |  |
| Economic status |  |  |  |  |  |  |
| low | 8 | (2.9) |  | 3 | (2.8) | 0.535 |
| medium | 129 | (46.9) |  | 44 | (40.7) |  |
| high | 138 | (50.2) |  | 61 | (56.5) |  |
| Place of residence (thousand citizens) |  | |  |  | |  |
| village | 84 | (30.6) |  | 27 | (25.0) | 0.062 |
| <50 | 57 | (20.7) |  | 24 | (22.2) |  |
| 50-100 | 29 | (10.6) |  | 4 | (3.7) |  |
| >100 | 105 | (38.2) |  | 53 | (49.1) |  |
| *n* | 275 | |  | 54 | |  |
| Education level |  |  |  |  |  |  |
| primary/vocational | 59 | (21.5) |  | 6 | (11.1) | 0.204 |
| high/technical | 111 | (40.4) |  | 26 | (48.2) |  |
| higher education | 105 | (38.2) |  | 22 | (40.7) |  |

**Supplementary Table S1.** Baseline characteristics of included and excluded participants. Notes: Values are presented as median (Me) and interquartile range (IQR) or number (%). BMI, body mass index; WC, waist circumference. WtHR, waist-to-height ratio; MUAC, mid-upper-arm muscle circumference; FM, total body fat mass; %FM, percentage of total body fat mass; ASM, Appendicular Skeletal Muscle Mass. *P* was obtained using Kruskal-Wallis with Dunn post-hoc test for continuous variables and chi-square or Fisher exact test for categorical variables.

| **Nutrient cut-offs** | **Total** | | **Skeletal Muscle Mass Index** | | | | | | ***P*** |
| --- | --- | --- | --- | --- | --- | --- | --- | --- | --- |
|  |  |  | **Bottom tertile** | | **Middle tertile** | | **Upper tertile** | |  |
|  | ***n*** | **%** | ***n*** | **%** | ***n*** | **%** | ***n*** | **%** |  |
| *n* | 275 |  | 90 |  | 93 |  | 92 |  |  |
| Protein <EAR | 63 | 22.9 | 11 | 12.2 | 22 | 23.7 | 30 | 32.6 | 0.005 |
| Total Fat >35%E | 208 | 75.9 | 66 | 73.3 | 75 | 80.6 | 67 | 72.8 | 0.383 |
| Fiber <AI | 256 | 93.1 | 87 | 96.7 | 88 | 94.6 | 81 | 88.0 | 0.056 |
| Calcium <EAR | 179 | 65.1 | 69 | 76.7 | 59 | 63.4 | 51 | 55.4 | 0.010 |
| Phosphorus <EAR | 10 | 3.6 | 5 | 5.6 | 5 | 5.4 | 0 | 0.0 | 0.073 |
| Magnesium <EAR | 201 | 73.1 | 68 | 75.6 | 72 | 77.4 | 61 | 66.3 | 0.190 |
| Zinc <EAR | 64 | 23.3 | 24 | 26.7 | 25 | 26.9 | 15 | 16.3 | 0.153 |
| Cooper <EAR | 40 | 14.5 | 18 | 20.0 | 11 | 11.8 | 11 | 12.0 | 0.202 |
| Potassium <EAR | 246 | 89.5 | 89 | 98.9 | 93 | 100 | 87 | 94.6 | 0.028 |
| Vitamin A <EAR | 63 | 22.9 | 27 | 30.0 | 25 | 26.9 | 11 | 12.0 | 0.008 |
| Vitamin E <AI | 126 | 45.8 | 47 | 52.2 | 44 | 47.3 | 35 | 38.0 | 0.149 |
| Vitamin B_1_ <EAR | 124 | 45.1 | 39 | 43.3 | 46 | 46.5 | 39 | 43.4 | 0.577 |
| Vitamin B_2_ <EAR | 43 | 15.6 | 19 | 21.1 | 15 | 16.1 | 9 | 9.8 | 0.108 |
| Niacin <EAR | 56 | 20.4 | 25 | 27.8 | 21 | 22.6 | 10 | 10.9 | 0.015 |
| Vitamin B_6_ <EAR | 45 | 16.4 | 21 | 23.3 | 16 | 17.2 | 8 | 8.7 | 0.027 |
| Vitamin C <EAR | 239 | 86.9 | 80 | 88.9 | 81 | 87.1 | 78 | 84.8 | 0.712 |

**Supplementary Table S2.** Subjects with inadequate nutrient intakes by bottom and upper tertiles of skeletal muscle mass index. Values are presented as number (%). Cut-off points were established according to Polish dietary recommendations. EAR, Estimated Average Requirements; AI, Adequate Intake; E, Energy. *P* was obtained using chi-square test or Fisher exact test.

| **Cut-offs for nutrient intakes** | **Detailed criteria for female and age-groups** |
| --- | --- |
| Protein <EAR | ≥19 years: <0.73 g/kg BW/d |
| Fat >35%E | ≥19 years: >35%E |
| Fiber <AI | 31-50 years: <25g/d  51-65 years: <25g/d |
| Ca <EAR | 31-50 years: <800 mg/d  51-65 years: <1000 mg/d |
| P <EAR | 31-50 years: <580 mg/d  51-65 years: <580 mg/d |
| Mg <EAR | 31-50 years: <265 mg/d  51-65 years: <265 mg/d |
| Zn <EAR | 31-50 years: <6.8 mg/d  51-65 years: <6.8 mg/d |
| Cu <EAR | 31-50 years: <0.7 mg/d  51-65 years: <0.7 mg/d |
| K <AI | 31-50 years: <3500mg/d  51-65 years: <3500 mg/d |
| Vitamin A <EAR | ≥19 years: <500 µg/d as retinol equivalent |
| Vitamin E <AI | ≥19 years: <8 mg/d as alpha-tocopherol equivalent |
| Vitamin B_1_ <EAR | ≥19 years: <0.9 mg/d |
| Vitamin B_2_ <EAR | ≥19 years: <0.9 mg/d |
| Niacin <EAR | ≥19 years: <11 mg/d |
| Vitamin B_6_ <EAR | 31-50 years: <1.1 mg/d  51-65 years: <1.3 mg/d |
| Vitamin C <EAR | ≥19 years: <60 mg/d |

**Supplementary Table S3.** Cut-off points used to identify the subjects with inadequate nutrient intakes. Established according to Polish dietary recommendations. EAR, Estimated Average Requirements. AI, Adequate Intake ; %E, percentage of energy
